# The role of isolation on contrasting phylogeographic patterns in two cave crustaceans

**DOI:** 10.1186/s12862-017-1094-9

**Published:** 2017-12-07

**Authors:** Jorge L. Pérez-Moreno, Gergely Balázs, Blake Wilkins, Gábor Herczeg, Heather D. Bracken-Grissom

**Affiliations:** 1 Department of Biological Sciences, Florida International University - Biscayne Bay Campus, 3000 NE 151 St., North Miami, FL 33181 USA; 2 Department of Systematic Zoology and Ecology, Eötvös Loránd University, Pázmány Péter Sétány 1/C, Budapest, H-1117 Hungary

**Keywords:** Adaptation, Biospeleology, Exaptation, Evolution, Phylogenetics, Subterranean, Troglomorphy

## Abstract

**Background:**

The underlying mechanisms and processes that prompt the colonisation of extreme environments, such as caves, constitute major research themes of evolutionary biology and biospeleology. The special adaptations required to survive in subterranean environments (low food availability, hypoxic waters, permanent darkness), and the geographical isolation of caves, nominate cave biodiversity as ideal subjects to answer long-standing questions concerning the interplay amongst adaptation, biogeography, and evolution. The present project aims to examine the phylogeographic patterns exhibited by two sympatric species of surface and cave-dwelling peracarid crustaceans (*Asellus aquaticus* and *Niphargus hrabei*), and in doing so elucidate the possible roles of isolation and exaptation in the colonisation and successful adaptation to the cave environment.

**Results:**

Specimens of both species were sampled from freshwater hypogean (cave) and epigean (surface) habitats in Hungary, and additional data from neighbouring countries were sourced from Genbank. Sequencing of mitochondrial and nuclear loci revealed, through haplotype network reconstruction (TCS) and phylogenetic inference, the genetic structure, phylogeographic patterns, and divergence-time estimates of *A. aquaticus* and *N. hrabei* surface and cave populations. Contrasting phylogeographic patterns were found between species, with *A. aquaticus* showing strong genetic differentiation between cave and surface populations and *N. hrabei* lacking any evidence of genetic structure mediated by the cave environment. Furthermore, *N. hrabei* populations show very low levels of genetic differentiation throughout their range, which suggests the possibility of recent expansion events over the last few thousand years.

**Conclusions:**

Isolation by cave environment, rather than distance, is likely to drive the genetic structuring observed between immediately adjacent cave and surface populations of *A. aquaticus,* a predominantly surface species with only moderate exaptations to subterranean life. For *N. hrabei*, in which populations exhibit a fully ‘cave-adapted’ (troglomorphic) phenotype, the lack of genetic structure suggests that subterranean environments do not pose a dispersal barrier for this surface-cave species.

**Electronic supplementary material:**

The online version of this article (10.1186/s12862-017-1094-9) contains supplementary material, which is available to authorized users.

## Background

One of the major recurring themes in evolutionary biology and ecology is discerning the drivers of genetic differentiation and diversity among populations, and their interplay with the environment. These patterns can often be associated to a wide array of geographic and environmental factors that influence population differentiation by means of both adaptive (i.e. selection) and non-adaptive (i.e. genetic drift) processes. As it is often observed in natural systems, reduced gene flow due to geographic distance can often result in distinct patterns of genetic differentiation across a spatial continuum [1]. However, other factors besides geographic distance often affect gene-flow between populations. Biotic and abiotic interactions can impact gene-flow through a variety of mechanisms (such as local adaptation, selection against immigrants, and biased dispersal), which result in populations being isolated by environmental differences [2–4]. Isolation by environment can be identified as a driver for observed patterns of genetic differentiation when there is a positive correlation between genetic and environmental differentiation, but no correlation between genetic differentiation and spatial distances between populations (the latter being an indication of isolation by distance) [4, 5]. It is important to note, however, that isolation by distance and isolation by environment are not mutually exclusive and their effects might be particularly challenging to disentangle when environmental variables and geographic distances co-vary [3]. Caves and other subterranean habitats show marked environmental differences with adjacent surface ecosystems with a sharp boundary, most notably the absence of light and associated ecological and biogeochemical conditions. Such habitat differences can constitute significant barriers to gene flow and population connectivity, which in turn lead to high levels of genetic differentiation even at relatively small spatial scales [6, 7].

In addition to how genetic diversity is distributed across distributional space, the underlying mechanisms and processes that prompt the colonisation of extreme environments, and more specifically caves, constitute one of the major research themes of evolutionary biology [8–10]. There are two major hypotheses generally regarded to explain the transition from surface to subterranean habitats. The adaptive shift hypothesis suggests that the colonisation of subterranean habitats is a result of founder populations actively expanding into and colonising new niches [11], rather than by accidental stranding and persistence in the aphotic zones [12, 13]. The ability of a species to successfully colonise these extreme environments, however, might be mediated by ecological filtering and thus requires specific exaptations to life in darkness [9, 14–16]. Possible exaptations to cave life include morphological (e.g. reduced dependence on vision, elongation of body and appendages) and physiological (e.g. tolerance to oligoxic conditions) characteristics that are already present in numerous species inhabiting benthic and interstitial ecosystems [17–19]. On the other hand, the climatic relict hypothesis states that a species may be forced to adapt to cave life as a result of environmental change that results in uninhabitable conditions on the surface (e.g. glaciation events) [18, 20, 21]. The actual mechanisms that gave rise to contemporary cave populations are likely to be a combination of both processes, and continue to be a subject of investigation. The estimation of phylogenetic relationships from genetic data of cave-dwellers offers the possibility of elucidating the mechanisms and processes that eventually lead to cave colonisations and the persistence of cave populations. This is especially the case when genetic data of both surface and cave-dwelling organisms are coupled with their present-day geographic distributions to infer ancient events (e.g. [22]). However, to fully understand the mechanisms behind cave colonisation events through present-day phylogeographic patterns it is imperative to incorporate approaches that consider the environment and ecology of the species under study, and therefore the underlying factors that ultimately drive their evolution.

**Fig. 1:**
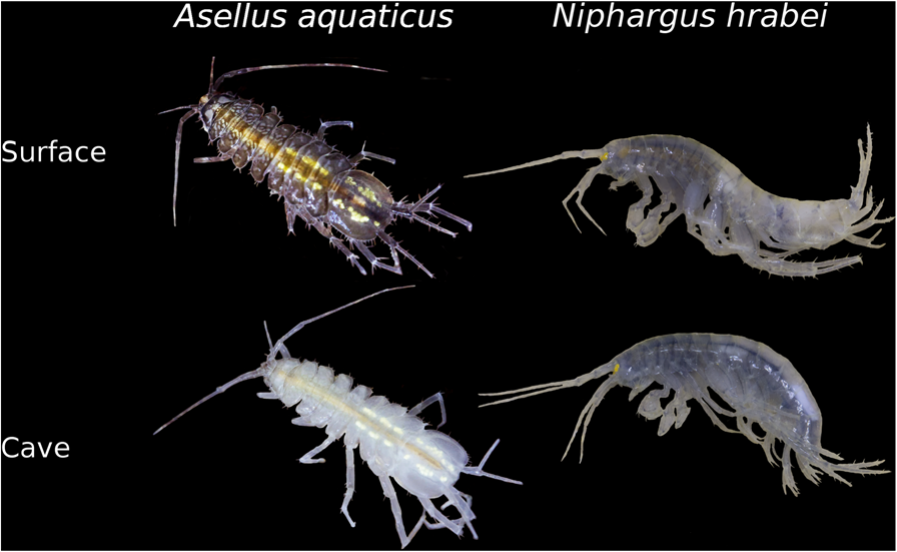
*Asellus aquaticus* displays contrasting phenotypes in and out of the cave, while *Niphargus hrabei* exhibits the same phenotype in both environments

The isopod *Asellus aquaticus* and the amphipod *Niphargus hrabei* are two aquatic crustacean species that serve as ideal models to explore questions regarding the colonisation, barriers to gene-flow, and evolution of cave fauna. *Asellus aquaticus* is a widespread species of freshwater isopod commonly found in surface waters throughout Europe [23]. This species is also known to occasionally colonise caves where its populations exhibit “troglomorphic” phenotypes [23, 24]. Troglomorphy can be defined as the set of morphological, physiological, and behavioural characteristics associated with a species transition to life in caves (e.g. enlarged sensory and ambulatory appendages, lack of pigmentation, loss of vision, etc. [25–28]. Contrastingly, the amphipod species *N. hrabei* is an atypical representative of an almost exclusively cave-dwelling genus that has escaped the confines of the subterranean environment to colonise surface habitats. Its distribution spans an extensive area of central and eastern Europe with geographical ranges of up to 1300 km [29], where it lives in sympatry with *A. aquaticus* (e.g. in the Danube River and its floodplains). Observations suggests that *N. hrabei* populations are troglomorphic throughout its distribution in both caves and surface waters (Fig. 1; [28]), perhaps due to the ancient cave-origin of the genus *Niphargus*. The disposition to inhabit both surface and cave environments, geographical distributions, and life-history characteristics of these two crustacean species make them ideal study organisms to disentangle the effects of isolation by distance and/or isolation by environment and to reveal the mechanisms and processes at play during cave colonisation.

The present study examines the phylogeographic patterns exhibited by sympatric surface and cave-dwelling populations of *A. aquaticus* and *N. hrabei*. We aim to test the hypothesis that isolation by environment drives the patterns of genetic differentiation of surface-exapted *A. aquaticus*, but not those of cave-exapted *N. hrabei,* for which isolation by distance is the expected mechanism. The Molnár János thermal cave system and the immediately adjacent Malom Lake (Budapest, Hungary) provide a perfect natural experiment to address questions of cave colonisation, adaptation, and population differentiation. Despite marked environmental differences between hypogean and epigean habitats, both localities are inhabited by the species under study and are hydrologically interconnected with the Danube River Basin, one of the largest and most species-rich natural floodplains in Europe [30, 31]. Therefore, the patterns of genetic differentiation that emerge from this system will allow for a better understanding of the effects of isolation (distance and/or environment) and possible roles of exaptations in the evolution of these cave and surface populations.

## Methods

### Sample collection

**Fig. 2:**
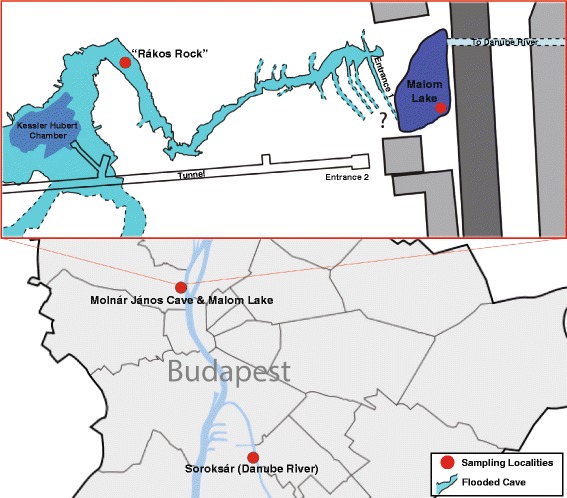
Schematic illustration of our thee main sampling localities within Budapest, Hungary. Red circles indicate exact sites within Molnár János Cave (Rákos Rock) and surface environments (Malom Lake and Danube River [Soroksár])

Specimens were sampled from three main sites in Budapest, Hungary: The Molnár János thermal cave system, the adjacent thermal Malom Lake, and the Soroksár branch of the Danube River (Fig. 2). The three sites are interconnected hydrologically and the two study species (*Asellus aquaticus* and *Niphargus hrabei*) inhabit all the sites. Additional specimens of *A. aquaticus* were sourced from other locations in Hungary (Table 1) and sequence data for *N. hrabei* from neighbouring countries were obtained from GenBank to aid in the analyses [29]. All of the samples were collected using a “Sket bottle” [32] and preserved individually in 99% ethanol for subsequent molecular analyses. All of the samples employed by this study are housed in the Florida International Crustacean Collection (FICC; North Miami, FL, USA). Additional metadata associated with each specimen is securely stored in the collection’s curated electronic database.

**Table 1:** Specimens, locations, and type of habitat in which the crustacean populations were sampled

Species	*N*	Locality	Coordinates	Habitat
*Asellus aquaticus*	20	Soroksár, Budapest, Hungary	47.4360697 N, 19.0878143 E	Epigean
	20	Molnár János Cave, Budapest, Hungary	47.5181846 N, 19.036064 E	Hypogean
	20	Malom Lake, Budapest, Hungary	47.5181167 N, 19.036075 E	Epigean
	4	Lipót, Hungary	47.86316 N, 17.458875 E	Epigean
	6	Polgár, Hungary	47.869443 N, 21.200598 E	Epigean
	6	Balatonfenyves, Hungary	46.65515 N, 17.498538 E	Epigean
	10	Cserdi, Hungary	46.06575 N, 17.991012 E	Epigean
*Niphargus hrabei*	20	Soroksár, Budapest, Hungary	47.4360697 N, 19.0878143 E	Epigean
	18	Molnár János, Budapest Hungary	47.5181846 N, 19.036064 E	Hypogean
	20	Malom Lake, Budapest Hungary	47.5181167 N, 19.036075 E	Epigean
^*a*^ *Niphargus* sp. nov.	13	Molnár János, Budapest, Hungary	47.5181846 N, 19.036064 E	Hypogean
^*a*^ *Niphargus forroi*	2	Diabáz Cave, Nagyvisnyo, Hungary	48.08809 N, 20.46627 E	Hypogean

^a^Used as outgroups for the analyses

### DNA extraction and amplification

Genomic DNA was extracted from each specimen’s pereiopods and/or antennae using the commercially available QIAGEN DNeasy Blood and Tissue Kit (Cat. No. 69506). Several mitochondrial and nuclear loci were selected in order to maximise the resolution at the scale of interest (population level). Specifically, for *A. aquaticus* the loci chosen were: two mitochondrial ribosomal genes (*12S* and *16S*), a mitochondrial protein-coding gene (cytochrome c oxidase subunit I, *COI*), and a ‘Numt’ (nuclear mitochondrial DNA segment) [33, 34] of NADH dehydrogenase 2 (hereby referred to as *PseudoND2*). For *N. hrabei* the sequenced loci included: a mitochondrial ribosomal gene (*16S*), a mitochondrial protein-coding gene (cytochrome c oxidase subunit I, *COI*), a nuclear ribosomal gene (internal transcribed spacer, *ITS*), and a nuclear protein-coding gene (*NaK*). These loci have proved to be useful in inferring intra- and interspecific relationships across the subphylum Crustacea (*12S*, *16S*, *COI*, *ITS*, *NaK*) [35–39] or were specifically targeted to increase population-level resolution (*PseudoND2*) [34]. Polymerase Chain Reaction (PCR) amplifications were performed in reactions containing DNA template, sterile non-DEPC treated water, forward and reverse primers, and of GoTaq® Green Master Mix (Promega, M712). The thermal cycling profiles consisted of an initial denaturing step of 1 min at 94 °C, followed by 35–40 cycles of 30 s at 94 °C, annealing for 30s at 48°-62 °C (depending on primer set and species, Additional file 1), 1 min extension at 72 °C, and then a final extension of 7 min at 72 °C. PCR products were sent to Beckman Coulter Genomics (Danvers, MA, USA) for amplicon purification using solid-phase reversible immobilisation (SPRI) beads, and subsequent sequencing reactions using BigDye Terminator v3.1. Post reaction dye terminator removal was done using Agencourt CleanSEQ, after which both forward and reverse strands were sequenced on an Applied Biosystems PRISM 3730xl DNA Analyzer.

### Data preparation and analyses of selection

The obtained sequences were visually inspected, quality trimmed, and cleaned manually with Geneious 8.0 [40]. Sequences from specimens heterozygous at nuclear loci were phased with PHASE v2.1 [41, 42] and SeqPHASE [43]. In instances where haplotype reconstruction during phasing resulted in more than one pair of possible sequences, pairs with the highest posterior probabilities were retained for subsequent analyses. To further verify the appropriateness of the chosen loci for phylogenetic inference, tests for selection in protein-coding loci were conducted with MEGA 7 [44]. Due to the small number of substitutions in most of the dataset, Fisher’s exact test of neutrality was chosen as the preferred option to detect evidence of selection [44, 45]. For this purpose, synonymous and non-synonymous substitutions were estimated using the Nei-Gojobori method [46].

### Haplotype network reconstruction

Haplotype networks were subsequently built in PopArt 1.7 [47] using TCS (Templeton-Crandall-Sing) Networks [48]. Haplotype networks allow for informative visualisation of genealogical information at shallow divergence levels. Although several methodologies exist for constructing these networks, TCS was chosen for its effectiveness at recovering accurate population-scale phylogeographic patterns even when genetic differentiation is low (e.g. [49–52]). Subsequent to haplotype network reconstruction, the relative frequencies of the mitochondrial haplotypes identified were plotted on maps to visualise their geographic distributions in an intuitive manner.

### Phylogenetic analyses

Individual and concatenated gene trees were estimated using Maximum Likelihood (GARLI 2.01 [53]) and Bayesian inference (MrBayes 3.2.6 [54]) methods as implemented in the CIPRES portal [55], after using PartitionFinder v1.1.1 [56] to identify the best-fit models of molecular evolution and partitioning schemes for the dataset (Additional file 1). Maximum Likelihood phylogenetic trees were reconstructed with an initial search for the best tree, using 10 parallel runs via GARLI 2.01. Additionally, 10,000 bootstrap replicates were generated in 40 independent runs to assess nodal support of the best tree. All ML trees were then summarised with a 95% consensus rule and annotated using the SumTrees.py python script from the DendroPy library [57]. Bayesian phylogenetic trees were inferred with the same partitioning scheme as in the ML analyses. MrBayes 3.2.6 was executed with two independent runs, each consisting of 4 chains running for 10 M generations. The MCMC run was sampled every 1000 generations, and a relative burn-in frequency of 25% was set for accurate posterior sampling. After assessing for convergence (Tracer v1.6 [58], the SumTrees.py script was again invoked to extract the Maximum Clade Credibility Tree (MCCT) and to annotate the phylogenetic trees’ nodal support as posterior probabilities. Further, population trees were estimated under a multi-locus coalescent model using *BEAST (BEAST 2.4.0 [59]). Intraspecific divergence times of *A. aquaticus* and *N. hrabei* populations were concurrently estimated using molecular clock rate calibrations for peracarid crustaceans’ *COI* (1.25% of substitutions per site per million years [60]; and between 0.34% and 0.76% of substitutions per site per Myr [61]), which were previously estimated for taxa closely related to our species of study (Stenasellidae and Niphargiidae). All of *BEAST and BEAST analyses were run in triplicate at Florida International University’s High Performance Computing cluster (Panther) for 200 M generations after which they were assessed for convergence using Tracer v1.6. The *BEAST speciation models for which there was no evidence for convergence were discarded. The runs using a Yule model of speciation as a tree prior with a strict molecular clock calibration were retained for subsequent analyses. After discarding 25% of the sampled trees as burn-in, nodal support was annotated as posterior probabilities on the MCCT of each population tree analysis (TreeAnnotator; [59]. For each species, population trees that included divergence time estimates were plotted using the *geoscale.phylo* function of the R package ‘strap’ [62]. These were then georeferenced on precomputed maps with custom scripts that made use of the R package ‘phytools’ [63].

Genealogical Sorting Indices (*gsi*) were calculated using the R package ‘genealogicalSorting’ to quantify intraspecific lineage divergence, which allowed for the evaluation of monophyly in each population [64, 65]. Lastly, a modified approach to calculate *gsi* (pairwise-gsi or *pwgsi*) was used to independently quantify the divergence of every population-pair [66]. This modified approach thus prevented possible bias and false-positives that could have arisen as by-products of the trees’ topologies outside the main groups of interest [66]. Outgroup populations of *N. hrabei* were excluded from this final analysis due to sample size requirements of the *pwgsi* approach. Nonetheless, this exclusion bears no impact on the comparisons amongst the target populations (Molnár János Cave, Malom Lake, and the Danube River’s Soroksár).

### Demographic history

Additionally, we sought to better understand the demographic histories of *A. aquaticus* and *N. hrabei*, as reflected in their sequence data, by estimating changes in their population sizes over time. For this purpose, we conducted Extended Bayesian skyline (EBS) analyses [67]) as implemented in BEAST [59]. Extended Bayesian skyline analyses allow for the incorporation of multi-locus datasets to estimate population history over time along with an assessment of the estimations’ uncertainty [67]. The parameters employed for these analyses were maintained as in the previous successful BEAST runs, with the exception of the priors associated to the species tree, which was set to “Coalescent Extended Bayesian Skyline”. These analyses were also run in triplicate at Florida International University’s High Performance Computing cluster (Panther) for 200 M generations after which they were assessed for convergence using Tracer v1.6. EBS run logs were subsequently combined, after discarding 25% as burn-in, and the demographic histories of both species were plotted using custom R scripts for ease of visualisation and further inferences.

## Results

### DNA sequences and data deposition

A total of ~1690 and ~2757 base pairs (bp) of nucleotide sequence data were recovered for *A. aquaticus* (81 sequences for *12S*, 83 for *16S*, 76 for *COI*, and 84 for *PseudoND2*) and *N. hrabei* (55 sequences for *16S*, 54 for *COI*, 51 for *ITS*, and 58 for *NaK*) respectively. All sequence data from this project were curated, annotated with their respective metadata, and deposited in the NCBI’s Genbank database to allow for their dissemination and future use by other researchers (See Additional file 1 for accession numbers).

### Testing for neutrality of selected loci

Fisher’s exact test of neutrality was employed to determine the suitability of the selected loci for phylogeographic inference by ensuring that they are not being subject to selective pressures. The probability (*P*) of rejecting the null hypothesis of strict-neutrality in favor of the alternative hypothesis of positive selection was larger than 0.05 for all loci in both species, and therefore not considered significant at an alpha value (α) of 5%. The chosen loci were therefore deemed suitable for subsequent analyses.

### Haplotype network reconstruction

**Fig. 3:**
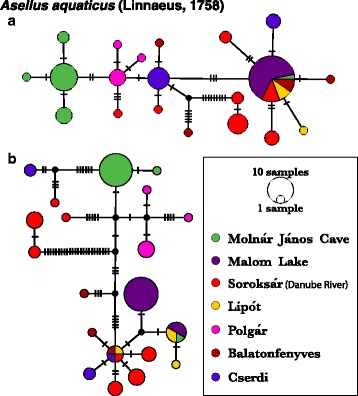
Haplotype networks of *Asellus aquaticus*: nuclear (**a**- PseudoND2) and mitochondrial (**b**- *16S*, *12S*, and *COI*) loci. Node diameter and annotation denote sample sizes, while hatch marks represent mutational steps between haplotypes. Colours represent sampling locality, as illustrated in the legend

**Fig. 4:**
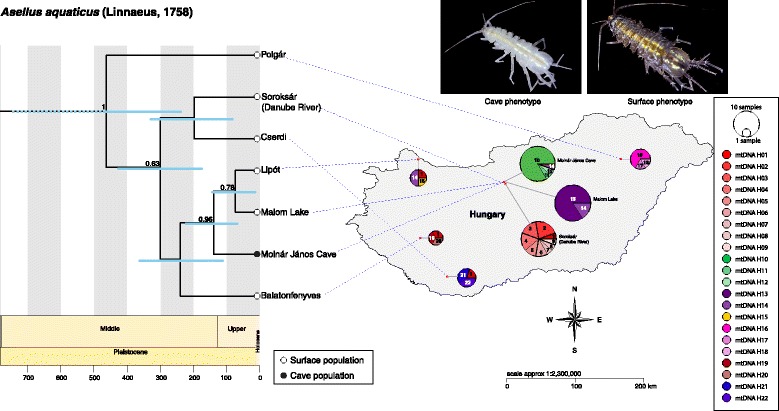
Divergence time estimates (x axis in thousands of years) of *Asellus aquaticus* populations (calculated with a multi-locus coalescent model in *BEAST; outgroups not shown) and the distribution of its populations with relative mtDNA haplotype frequencies throughout Hungary. Phylogenetic and population tree analyses support the inclusion of the cave phenotype as part of the species, but with evident population structuring as a result of the cave environment

**Fig. 5:**
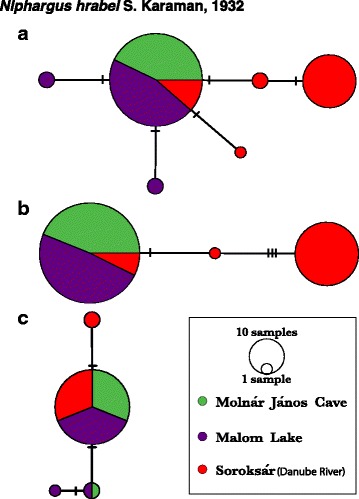
Haplotype networks of *Niphargus hrabei*: nuclear (**a**- *ITS*; **b**- *NaK*) and mitochondrial (**c**- *16S* and *COI*) loci. Node diameter and annotation denote sample sizes (alleles in the case of nuclear genes), while hatch marks represent mutational steps between haplotypes. Colours represent sampling locality, as illustrated in the legend

**Fig. 6:**
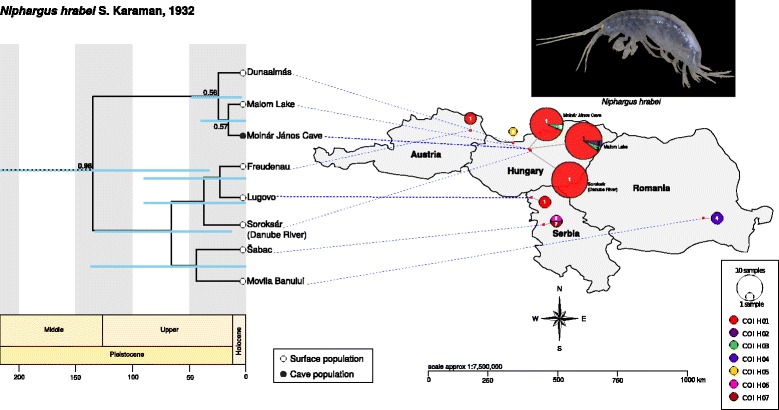
Divergence time estimates (x axis in thousands of years) of *Niphargus hrabei* populations (calculated with a multi-locus coalescent model in *BEAST; outgroups not shown) and the distribution of its populations with relative *COI* haplotype frequencies in our three main Hungarian sites and neighbouring populations. Phylogenetic and population tree analyses do not support any evident genetic structuring between cave and surface populations

Analyses of haplotype networks display evident genetic structuring in surface and cave *A. aquaticus,* with particularly distinct haplotypes differentiating Molnár János Cave’s population from Malom Lake’s despite their spatial proximity (Fig. 3). The haplotypes found in Molnár János Cave and Malom Lake are exclusive to each locality, with the exception of a single “surface-phenotype” cave specimen that shared a haplotype found in both Malom Lake and the western population of Lipót (Figs. 3, 4). This strong differentiation and lack of shared haplotypes between the cave’s and adjacent surface populations is not the case for *N. hrabei,* where Malom Lake’s and Molnár János Cave’s mtDNA haplotypes are the same and no genetic structuring is evident (Fig. 5). Mitochondrial haplotypes (*COI* and *16S*) of *N. hrabei* are not only closely related, but also found widely distributed throughout its range (Figs. 5, 6).

### Phylogeographic and genealogical sorting analyses

The patterns observed in the haplotype network reconstructions are reflected in and confirmed by the concatenated phylogenetic trees (tree-files are available in the figshare respository at:  https://doi.org/10.6084/m9.figshare.5660542.v1). The final Maximum Likelihood and Bayesian trees for each species are nearly identical, with the exception of a few unsupported nodes, and are concordant with the *BEAST population trees estimated with all the sequenced loci (*A. aquaticus*, Fig. 4; *N. hrabei*, Fig. 6; see Additional file 1 for evolutionary model selection details). Furthermore, these population trees show that cave and surface populations of *A. aquaticus* diverged from each other at least 60 k years ago (Table 2). There is no evidence for genetic structuring between cave and surface populations of *N. hrabei*, and the phylogenetic split between these is not supported. The Pairwise Genealogical Sorting Index (*pwgsi*) estimates follow patterns similar to those of the population tree topologies recovered (*A. aquaticus,* Table 3; *N. hrabei,* Table 4). In *A. aquaticus*, the distinction between the Molnár János Cave population and the adjacent Malom Lake is clear despite its proximity (*pwgsi* = 1.00, *p* < 0.001), with the former having higher affinities to the south-western population of Balatonfenyves (*pwgsi* = 0.22, *p* < 0.001). The *pwgsi* between Molnár János Cave’s and Malom Lake’s *N. hrabei* populations on the other hand, provides no evidence of genealogical differentiation between these (*pwgsi* = 0.14, *p* = 0.52). Nevertheless, both populations display a statistically significant but modest degree of reciprocal monophyly when compared to the next geographically closest population, the Soroksár branch of the Danube River (*pwgsi* = 0.46 and 0.48 respectively, both *p* < 0.001).

**Table 2:** Divergence time estimations between Molnár János Cave and phylogenetically closest surface populations of the peracarid crustaceans under study (thousands of years [95% H.P.D.])

	Molecular Clock Rate	
Species	Ketmaier et al. (2003)	Lefébure et al. (2006)
*Asellus aquaticus*	60.81 (28.68, 96.53)	139.21 (67.07, 222.26)
*Niphargus hrabei*	Surface/cave split not supported	Surface/cave split not supported

**Table 3:** Pairwise Genealogical Sorting Index estimates for each population pair of *Asellus aquaticus*

		Bayesian Phylogeny		Maximum Likelihood Phylogeny	
Population 1	Population 2	*pwgsi*	*p*-value	*pwgsi*	*p*-value
Molnár János Cave	Soroksár (Danube)	0.71	< 0.001	0.68	< 0.001
Molnár János Cave	Malom Lake	1.00	< 0.001	1.00	< 0.001
Molnár János Cave	Lipót	0.46	< 0.001	0.35	< 0.001
Molnár János Cave	Polgár	0.89	< 0.001	0.89	< 0.001
Molnár János Cave	Balatonfenyves	0.22	< 0.001	0.22	< 0.001
Molnár János Cave	Cserdi	0.56	< 0.001	0.57	< 0.001
Soroksár (Danube)	Malom Lake	1.00	< 0.001	1.00	< 0.001
Soroksár (Danube)	Lipót	0.49	< 0.001	0.43	< 0.001
Soroksár (Danube)	Polgár	0.89	< 0.001	0.89	< 0.001
Soroksár (Danube)	Balatonfenyves	0.17	0.003	0.13	0.001
Soroksár (Danube)	Cserdi	0.46	< 0.001	0.43	< 0.001
Malom Lake	Lipót	1.00	< 0.001	1.0	< 0.001
Malom Lake	Polgár	1.00	< 0.001	1.0	< 0.001
Malom Lake	Balatonfenyves	1.00	< 0.001	1.0	< 0.001
Malom Lake	Cserdi	1.00	< 0.001	1.0	< 0.001
Lipót	Polgár	1.00	< 0.001	1.0	< 0.001
Lipót	Balatonfenyves	0.38	< 0.001	0.23	< 0.001
Lipót	Cserdi	0.47	< 0.001	0.47	< 0.001
Polgár	Balatonfenyves	0.78	< 0.001	0.78	< 0.001
Polgár	Cserdi	0.83	< 0.001	0.83	< 0.001
Balatonfenyves	Cserdi	0.15	0.012	0.15	< 0.001

*P*-values assess significance that exclusive ancestry observed for every population pair is greater than that which would be observed at random

**Table 4:** Pairwise Genealogical Sorting Index estimates for each population pair of *Niphargus hrabei*

		Bayesian Phylogeny		Maximum Likelihood Phylogeny	
Population 1	Population 2	*pwgsi*	*p*-value	*pwgsi*	*p*-value
Soroksár (Danube)	Molnár János Cave	0.46	<0.001	0.15	<0.001
Soroksár (Danube)	Malom Lake	0.48	<0.001	0.18	<0.001
Molnár János Cave	Malom Lake	0.14	0.521	0.06	0.005

*P*-values assess significance that exclusive ancestry observed for every population pair is greater than that which would be observed at random

### Demographic history

**Fig. 7:**
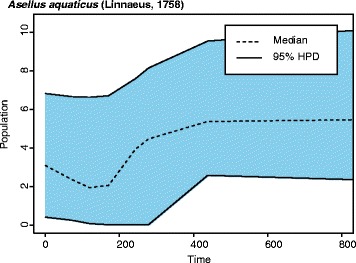
An Extended Bayesian Skyline Plot illustrates the demographic history of the sampled Hungarian *Asellus aquaticus* populations over the last 800,000 years. The x-axis represents time before present in thousands of years, while the y-axis denotes the estimated population size (θ) assuming a generation time of one year

**Fig. 8:**
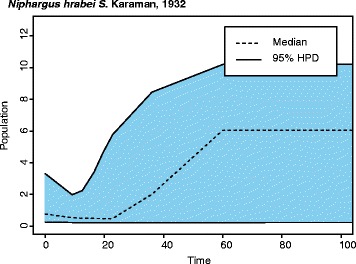
An Extended Bayesian Skyline Plot of *Niphargus hrabei*’s demographic history, from 100,000 years ago to today, depicts a possible genetic bottleneck at approximately 10,000 years ago. The x-axis represents time before present in thousands of years, while the y-axis denotes the estimated population size (θ) assuming a generation time of one year

We conducted Extended Bayesian Skyline (EBS) analyses [67]), as implemented in BEAST [59], to evaluate the demographic history of our two study species and investigate if there is any evidence of possible climate-associated population changes. The EBS plot for *A. aquaticus* shows a gradual population contraction reaching a minimum approximately between 100 and 200 thousand years ago and a gradual recovery thereafter (Fig. 7). Contrastingly, EBS analyses for *N. hrabei* point to a sharper decline beginning at a later date (~ 60 thousand years ago). The 95% H.P.D. interval suggests that an evident population bottleneck followed by a rapid expansion occurred approximately 10 thousand years before present (Fig. 8), roughly corresponding to the end of the Würm glaciation (~ 11,700 years ago).

## Discussion

The most intriguing finding of the present study is the population differentiation between cave and surface populations of *Asellus aquaticus*, a pattern which is not reflected in *Niphargus hrabei*. Here, we will discuss the phylogeographic patterns in the light of alternate isolation mechanisms (geographic distance *vs.* environment). Second, we focus on the Molnár János Cave system, and discuss its potential role as a climatic refugium together with the role of exaptation in successful cave colonisation. Lastly, we conclude by illustrating future possibilities and directions for research in this emergent study system.

### Contrasting phylogeographies: Isolation by distance, environment or both?

Our first objective was to resolve the phylogeographic patterns between sympatric surface and cave populations of two crustaceans in order to investigate if the cave environment is acting as a barrier for dispersal and connectivity of populations. The *A. aquaticus* populations throughout Hungary are genetically diverse with each population being comprised mostly by distinct, but closely related, mitochondrial haplotypes exclusive to their respective localities. Phylogenetic and population tree analyses do provide strong statistical support for the genetic differentiation between Molnár János Cave’s subterranean population and its epigean counterparts. Our results further suggest that the observed genetic and phenotypic differentiation between cave and surface isopods result from the cave acting as an isolating environment. Nevertheless, despite this differentiation, the Molnár János Cave *A. aquaticus* population still falls well within the species range for *A. aquaticus* in a phylogenetic context (Fig. 4). Furthermore, the presence of a single haplotype (mtDNA H01) in all of the western Hungarian populations sampled for this study suggests that movement over large distances does occur, suggesting a smaller role for geographical distance (*vs.* environment) as a driver of genetic differentiation (Fig. 4). The relatively high haplotypic diversity found in the Danube River (Soroksár, in comparison to the other localities examined) further supports its role as a dispersal avenue for isopods inhabiting surface waters. Effective dispersal in surface environments, but not into the cave, is further evident in our phylogenetic analyses by the lack of nodal support for the differentiation of the surface populations.

Interestingly, movement of individuals from Malom Lake into the cave was detected by the sampling of a single isopod with both surface haplotype (mtDNA H14) and phenotype (Figs. 3 & 4). Personal observations confirm that, in rare instances, surface isopods can be found in the aphotic zone well within Molnár János Cave. However, surface isopods do not persist in the cave habitat and the lack of shared mitochondrial and nuclear haplotypes suggests infrequent or non-existent admixture between these two genetically close but phenotypically distinct populations (Figs. 3 & 4). A similar pattern was observed in Slovenian and Romanian *A. aquaticus* where no population connectivity was found between troglomorphic cave isopods and other nearby populations from surface waters [24]. This recurrent pattern could be explained by a variety of mechanisms ultimately driven by the environmental differences between subterranean and epigean habitats [2–4]. It is thus feasible that surface individuals who wander into the cave are outcompeted by the troglomorphic resident population (i.e. competitive exclusion) before successful breeding takes place, and/or that hybrid individuals are at a significant fitness disadvantage that prevents their genes from persisting in the cave population [3]. Investigating and understanding which exact mechanisms might be at play in the Molnár János Cave system undoubtedly constitutes an important question to address in future studies.

Unlike that of *A. aquaticus,* haplotype network reconstruction and population tree analyses of *N. hrabei* show no evidence of genetic structuring between surface and cave populations (Figs. 5 & 6). In fact, haplotypic diversity seems to be relatively low throughout its range (Fig. 6). The only phylogeographic pattern recovered is the segregation of Hungarian populations as a distinct clade (Fig. 6). However, geography does not explain the inclusion of Danube River (Soroksár) individuals within the clade comprised by the Austrian, Serbian, and Romanian specimens. Low genetic diversity, weak genetic structuring despite large geographical distances, and lack of statistical support for any of the populations sampled, suggest that this species’ modern populations resulted from a recent expansion event and do not provide any clear evidence for isolation by distance nor environment. This is further evidenced by the results of our EBS analyses that suggest a population bottleneck for *N. hrabei* at approximately 10 thousand years ago followed by a rapid expansion (Fig. 8). It is important to note that sampling for some of the localities examined was limited and this could have a negative effect on statistical support for those populations. Nevertheless, the three main target populations of the present study (Molnár János cave, Malom Lake and Danube River [Soroksár]) were sufficiently sampled to adequately evaluate the population structure of surface vs. adjacent cave populations. Therefore, for *N. hrabei*, whose populations exhibit a fully ‘troglomorphic’ phenotype in all environments, the lack of genetic structure at this scale suggests that subterranean environments do not pose a barrier for this species. Further analyses employing high-resolution data (e.g. genome-wide SNPs) from next-generation sequencing methodologies would undoubtedly be of advantage to clarify whether this lack of genetic structure is truly due to unimpeded movement in and out of the cave or a by-product of *N. hrabei*’s recent colonisation of the habitats under investigation [10].

### The Molnár János thermal cave system: A climatic refugium and a possible role for exaptation

Divergence-time estimates, calibrated with peracarid *COI* molecular clock rates [60, 61], place the divergence of *A. aquaticus* populations from Molnár János Cave and Malom Lake at approximately 60,000 to 140,000 years ago (Fig. 4). This relatively recent split falls within the Pleistocene, a period of time during which severe climatic changes associated with glaciation events impacted the geographical distributions of numerous taxa across the globe [21, 68]. Even though Hungary was not directly glaciated, hydrological changes in the region would have altered the viability of this crustacean’s surface populations and shifted their latitudinal distributions [21, 68].

Caves have been known to act as refuges during rapid environmental changes on the surface [21, 23, 69–71]. Molnár János Cave’s waters are exclusively fed by thermomineral springs with a constant water temperature (~ 24 °C), which increases its suitability as a refugium for organisms with exaptations that help them subsist in cave environments. Being a moderately exapted species, it is possible that *A. aquaticus* would have been able to take refuge in the cave where it remained isolated from surface populations. This isolation eventually resulted in the emergence of troglomorphic phenotypes via adaptive and neutral processes. Upon cessation of this isolation, it is possible that competitive exclusion prevented new and/or returning surface populations from successfully invading the cave and vice versa. This mechanism would be in accordance with the observed phylogeographic patterns. Extended Bayesian Skyline Plot analyses illustrate a population decline for *A. aquaticus* and the possibility of the aforementioned events occurring approximately 100–200 thousand years ago (Fig. 7). It is also possible that *A. aquaticus* may have had a constant food source independent from the surrounding surface environment, as bacterial communities in Molnár János Cave, upon which *A. aquaticus* feeds (pers. obs.), have been shown to thrive via chemoautotrophic processes [72].

*Niphargus hrabei* is likely to have colonised Molnár János Cave thousands of years later, as suggested by the divergence-time estimates (Fig. 6). *Niphargus hrabei* cave and surface populations appear to be panmictic and show no evidence of isolation by the cave environment or of competitive exclusion within the cave. They have successfully colonised the cave from surface populations and appear to have no limitations with dispersing from and into cave environments. *Niphargus hrabei*’s facility for dispersal and its exceptional adaptability to markedly different habitats is reflected by an atypical large distributional range [29]. This adaptability is also evidenced by its unimpeded presence despite putative competitors in Molnár János Cave: the isopod *A. aquaticus* and two other species of *Niphargus* that are yet to be described (pers. obs.). However, an alternative explanation would be that older populations of *N. hrabei* once inhabited the Molnár János Cave, but were outcompeted by the obligate cave-dwellers in absence of migrants from surface waters. In fact, our EBS analyses show an abrupt population decline with a bottleneck and subsequent expansion at approximately 10 thousand years ago (Fig. 8) that roughly coincides with the end of the Würm glaciation, event which took place approximately between 115,000 and 11,700 years ago [73]. The end of the Würm glaciation is known for great climatic variability [74] with major temperature fluctuations that played a significant role in shaping the modern distributional patterns of other European species ([73, 75, 76]). Nevertheless, niphargiid coexistence in other caves has also been previously explained by evolutionary and ecological processes such as niche differentiation [77]. Understanding the exact mechanisms by which these processes take place continues to be an important research theme in evolutionary biology and biospeleology.

### Conclusions

The Molnár János Cave system and its inhabitants serve as ideal models for phylogeographic and biospeleological studies in an evolutionary context. While the present study has provided significant insights into the phylogeographic histories of two species and their transition into and out of caves, important questions remain to be answered. Further analyses will greatly aid in the understanding of the exact causes of the observed patterns, as well as in the elucidation of the mechanisms by which exaptations have helped them thrive in such extreme environments. An integrative approach incorporating different sources of molecular data (e.g. genomic, transcriptomic, epigenetic, etc.) has been initiated and will be of definitive advantage to address these outstanding questions [10]. Advances in modern molecular methodologies will undoubtedly enable future high-resolution studies of the adaptive processes that underlie the contrasting phylogeographic patterns revealed by this study.

## Additional file

Additional file 1: Additional tables with information regarding evolutionary models selected for phylogenetic analyses, the genetic loci sequence and primers employed, accession numbers for data deposited in GenBank, and Genealogical Sorting Index estimates for all populations of *Asellus aquaticus* and *Niphargus hrabei*. (DOCX 56 kb)

## Abbreviations

DEPC: Diethylpyrocarbonate
DNA: Deoxyribonucleic acid
EBS: Extended Bayesian Skyline
FICC: Florida International Crustacean Collection
gsi: Genealogical Sorting Index
HPD: Highest Posterior Density
MCCT: Maximum Clade Credibility Topology
MCMC: Markov chain Monte Carlo
ML: Maximum Likelihood
mtDNA: Mitochondrial DNA
NCBI: National Center for Biotechnology Information
Numt: Nuclear mitochondrial DNA segment
PCR: Polymerase Chain Reaction
pwgsi: Pairwise Genealogical Sorting Index
SPRI: Solid Phase Reversible Immobilization
TCS: Templeton-Crandall-Sing
