# Supplementary material for: The role of isolation on contrasting phylogeographic patterns in two cave crustaceans
Source: BMC Evol Biol. 2017 Dec 7;17:247. doi: 10.1186/s12862-017-1094-9 (PMC5721366; doi:10.1186/s12862-017-1094-9)
Supplement: Additional file 1: — Additional tables with information regarding evolutionary models selected for phylogenetic analyses, the genetic loci sequence and primers employed, accession numbers for data deposited in GenBank, and Genealogical Sorting Index estimates for all populations of Asellus aquaticus and Niphargus hrabei. (DOCX 56 kb) [file 12862_2017_1094_MOESM1_ESM.docx]

Table S1. Best-fit models of evolution for each locus and phylogenetics software employed, as determined with PartitionFinder (Lanfear et al., 2012).

| **Species** | **Locus** | **BEAST** | **MrBayes** | **GARLI** |
| --- | --- | --- | --- | --- |
| *Asellus aquaticus* | 12S | HKY+G+X | HKY+G | TRN+G |
|  | 16S | HKY+G+X | HKY+G | TRN+G |
|  | COI | HKY+G+X | HKY+G | HKY+G |
|  | PseudoND2 | HKY+G+X | HKY+G | HKY+G |
| *Niphargus* spp. | 16S | HKY+G+X | HKY+G | HKY+G |
|  | COI | HKY+G+X | HKY+G | HKY+G |
|  | NaK | TRNEF+I | K80+G | TRNEF+I |
|  | ITS | HKY+G+X | K80+I+G | K80+G |

Table S2. Loci sequenced and primers used for PCR amplification. Optimal PCR annealing temperatures are shown for each locus.

| **Species** | **Locus** | **Forward Primer** | **Forward Primer  Sequence** | **Reverse Primer** | **Reverse Primer  Sequence** | **Annealing Temp. °C** | **Reference** |
| --- | --- | --- | --- | --- | --- | --- | --- |
| *Asellus aquaticus* | 12S | 12S_F | GAAACCAGGATTAGATACCC | 12S_1R | AGCGACGGGCGATATGTAC | 60 | Buhay et al., 2007. |
|  | 16S | 16S_L | CGCCTGTTTAACAAAAACAT | 16S_H | CCGGTCTGAACTCAGATCACGT | 51 | Simon et al., 1994. |
|  | COI | LCOI-1490 | GGTCAACAAATCATAAAGATATTG | HCOI-2198 | TAAACTTCAGGGTGACCAAAAAATCA | 46 | Folmer et al., 1994 |
|  | PseudoND2 | PseudoND2_F | TCAGGGCTTGAGTGGGCCTAG | PseudoND2_R | CCCTACAGCTCCTAAGGTGGC | 62 | This study |
|  |  |  |  |  |  |  |  |
| *Niphargus* spp. | 16S | 16S_L2 | TGCCTGTTTATCAAAAACAT | 16S_1472R | AGATAGAAACCAACCTGG | 48 |  |
|  |  | 16S_L9 | CGCCTGTTTATCAAAAACAT | “ | “ |  |  |
|  | COI | COI_Nith_F | AGGTGCTTGGTCTAGTGTACTAG | COI_Nith_R | GATAGGATCCCCACCACCTCTAG | 58 | This study |
|  | NaK | NaK_for-b | ATGACAGTTGCTCATATGTTGGTTT | NaK_rev2 | ATAGGGTGATCTCCAGTRACCAT | 52 | Tsang et al., 2008. |
|  | ITS | ITS_Niph1f | TCCGAACTGGTGCACTTAGA | ITS_Niph1r | TCCAAGCTCCATTGGCTTAT | 62 | Flot et al., 2010. |
|  |  | ITS_Niph2f | CGCTGCCATTCTCACACTTA | ITS_Niph2r | ACTCTGAGCGGTGGATCACT | Sequencing only | “ |
|  |  | ITS_Niph3f | AAGGCTATAGCTGGCGATCA | ITS_Niph3r | TCAGCGGGTAACCTCTCCTA | Sequencing only | “ |

Table S3. Sequence data obtained for the present study and their corresponding GenBank accession numbers.

| **Species/Locality** | **Sample ID** | **12S** | **16S** | **COI** | **PseudoND2** | **ITS** | **NaK** |
| --- | --- | --- | --- | --- | --- | --- | --- |
| *Asellus aquaticus* |  |  |  |  |  |  |  |
| Soroksár  (Danube River) | HBG2880 | MG205174 | MG205246 | MG548044 | MG205382 |  |  |
|  | HBG2881 | MG205175 | MG205247 | MG548050 | MG205383 |  |  |
|  | HBG2882 | MG205176 | MG205248 | MG548053 | MG205384 |  |  |
|  | HBG2883 | MG205177 | MG205249 | MG548061 | MG205385 |  |  |
|  | HBG2884 | MG205178 | MG205250 | MG548045 | MG205386 |  |  |
|  | HBG2885 | MG205179 | MG205251 | MG548065 | MG205387 |  |  |
|  | HBG2886 | MG205180 | MG205252 | MG548049 | MG205388 |  |  |
|  | HBG2887 | MG205181 | MG205253 | MG548093 | MG205389 |  |  |
|  | HBG2888 | MG205182 | MG205254 | MG548066 | MG205390 |  |  |
|  | HBG2889 | MG205183 | MG205255 | MG548064 | MG205391 |  |  |
|  | HBG2890 | MG205184 | MG205256 | MG548062 | MG205392 |  |  |
|  | HBG2891 | MG205185 | MG205257 | MG548119 | MG205393 |  |  |
|  | HBG2892 | MG205186 | MG205258 | MG548063 | MG205394 |  |  |
|  | HBG2893 | MG205187 | MG205259 | MG548046 | MG205395 |  |  |
|  | HBG2894 | MG205188 | MG205260 | MG548048 | MG205396 |  |  |
|  | HBG2895 | MG205189 | MG205261 | MG548067 | MG205397 |  |  |
|  | HBG2896 | MG205190 | MG205262 | MG548047 | MG205398 |  |  |
|  | HBG2897 | MG205191 | MG205263 | MG548051 | MG205399 |  |  |
|  | HBG2898 | MG205192 | MG205264 | MG548052 | MG205400 |  |  |
| Molnár János | HBG2919 | MG205193 | MG205265 | MG548096 | MG205401 |  |  |
|  | HBG2920 | MG205194 | MG205266 | MG548097 | MG205402 |  |  |
|  | HBG2921 | MG205195 | MG205267 | N/A | MG205403 |  |  |
|  | HBG2922 | MG205196 | MG205268 | MG548098 | MG205404 |  |  |
|  | HBG2923 | MG205197 | MG205269 | MG548099 | MG205405 |  |  |
|  | HBG2924 | MG205198 | MG205270 | MG548100 | MG205406 |  |  |
|  | HBG2925 | MG205199 | MG205271 | MG548101 | MG205407 |  |  |
|  | HBG2926 | MG205200 | MG205272 | MG548107 | MG205408 |  |  |
|  | HBG2927 | MG205201 | MG205273 | MG548111 | MG205409 |  |  |
|  | HBG2928 | MG205202 | MG205274 | MG548102 | MG205410 |  |  |
|  | HBG2929 | MG205203 | MG205275 | MG548112 | MG205411 |  |  |
|  | HBG2930 | MG205204 | MG205276 | MG548068 | MG205412 |  |  |
|  | HBG2931 | MG205205 | MG205277 | MG548103 | MG205413 |  |  |
|  | HBG2932 | MG205206 | MG205278 | MG548104 | MG205414 |  |  |
|  | HBG2934 | MG205207 | MG205279 | MG548108 | MG205415 |  |  |
|  | HBG2935 | MG205208 | MG205280 | MG548109 | MG205416 |  |  |
|  | HBG2936 | MG205209 | MG205281 | MG548110 | MG205417 |  |  |
|  | HBG2937 | MG205210 | MG205282 | MG548105 | MG205418 |  |  |
|  | HBG2938 | MG205211 | MG205283 | MG548106 | MG205419 |  |  |
| Malom Lake | HBG2859 | MG205212 | MG205284 | MG548069 | MG205420 |  |  |
|  | HBG2860 | MG205213 | MG205285 | MG548075 | MG205421 |  |  |
|  | HBG2861 | MG205214 | MG205286 | MG548076 | MG205422 |  |  |
|  | HBG2862 | MG205215 | MG205287 | MG548077 | MG205423 |  |  |
|  | HBG2863 | MG205216 | MG205288 | MG548078 | MG205424 |  |  |
|  | HBG2864 | MG205217 | MG205289 | MG548079 | MG205425 |  |  |
|  | HBG2865 | MG205218 | MG205290 | MG548088 | MG205426 |  |  |
|  | HBG2866 | MG205219 | MG205291 | MG548070 | MG205427 |  |  |
|  | HBG2867 | MG205220 | MG205292 | MG548089 | MG205428 |  |  |
|  | HBG2868 | MG205221 | MG205293 | MG548080 | MG205429 |  |  |
|  | HBG2869 | MG205222 | MG205294 | MG548081 | MG205430 |  |  |
|  | HBG2870 | MG205223 | MG205295 | MG548090 | MG205431 |  |  |
|  | HBG2871 | MG205224 | MG205296 | MG548071 | MG205432 |  |  |
|  | HBG2872 | MG205225 | MG205297 | MG548082 | MG205433 |  |  |
|  | HBG2873 | MG205226 | MG205298 | MG548083 | MG205434 |  |  |
|  | HBG2874 | MG205227 | MG205299 | MG548091 | MG205435 |  |  |
|  | HBG2875 | MG205228 | MG205300 | MG548084 | MG205436 |  |  |
|  | HBG2876 | MG205229 | MG205301 | MG548085 | MG205437 |  |  |
|  | HBG2877 | MG205230 | MG205302 | MG548086 | MG205438 |  |  |
|  | HBG2878 | MG205231 | MG205303 | MG548087 | MG205382 |  |  |
| Lipót | HBG3991 | N/A | MG205304 | MG548074 | MG205439 |  |  |
|  | HBG3992 | N/A | MG205305 | MG548072 | MG205440 |  |  |
|  | HBG3993 | MG205232 | MG205306 | MG548073 | MG205441 |  |  |
|  | HBG3994 | N/A | MG205307 | MG548058 | MG205442 |  |  |
| Polgár | HBG3995 | MG205235 | MG205308 | MG548113 | MG205443 |  |  |
|  | HBG3996 | MG205238 | MG205318 | MG548114 | MG205444 |  |  |
|  | HBG3997 | MG205236 | MG205309 | MG548115 | MG205445 |  |  |
|  | HBG3998 | MG205240 | MG205310 | MG548118 | MG205446 |  |  |
|  | HBG3999 | MG205239 | MG205311 | MG548117 | MG205447 |  |  |
|  | HBG4000 | MG205237 | MG205312 | MG548116 | MG205448 |  |  |
| Balatonfenyves | HBG4001 | N/A | MG205319 | MG548092 | MG205449 |  |  |
|  | HBG4002 | MG205245 | MG205327 | N/A | MG205450 |  |  |
|  | HBG4003 | N/A | MG205313 | MG548060 | MG205451 |  |  |
|  | HBG4004 | N/A | MG205314 | MG548054 | MG205452 |  |  |
|  | HBG4005 | MG205233 | MG205315 | MG548055 | MG205453 |  |  |
| Cserdi | HBG4007 | MG205234 | MG205316 | MG548059 | MG205455 |  |  |
|  | HBG4008 | MG205241 | MG205321 | N/A | MG205456 |  |  |
|  | HBG4009 | MG205242 | MG205322 | MG548094 | MG205457 |  |  |
|  | HBG4010 | N/A | MG205325 | N/A | MG205458 |  |  |
|  | HBG4012 | MG205243 | MG205326 | N/A | MG205460 |  |  |
|  | HBG4013 | N/A | MG205317 | MG548056 | MG205461 |  |  |
|  | HBG4015 | MG205244 | MG205324 | MG548095 | MG205463 |  |  |
|  | HBG4016 | N/A | MG205328 | MG548057 | MG205464 |  |  |
|  |  |  |  |  |  |  |  |
| *Niphargus* *hrabei* |  |  |  |  |  |  |  |
| Soroksár (Danube River) | HBG2900 |  | MG205330 | MG548127 |  | N/A | MG548180 |
|  | HBG2901 |  | MG205369 | MG548120 |  | MG205466 | MG548181 |
|  | HBG2902 |  | MG205367 | MG548131 |  | N/A | MG548186 |
|  | HBG2903 |  | MG205370 | MG548123 |  | MG205512 | MG548187 |
|  | HBG2904 |  | MG205371 | MG548146 |  | MG205501 | MG548188 |
|  | HBG2905 |  | MG205366 | MG548147 |  | MG205502 | MG548189 |
|  | HBG2906 |  | MG205372 | N/A |  | MG205503 | MG548190 |
|  | HBG2907 |  | MG205378 | MG548148 |  | N/A | MG548191 |
|  | HBG2908 |  | MG205379 | MG548149 |  | MG205504 | MG548198 |
|  | HBG2909 |  | MG205368 | MG548159 |  | MG205499 | MG548182 |
|  | HBG2910 |  | MG205373 | MG548132 |  | MG205505 | MG548192 |
|  | HBG2911 |  | N/A | MG548150 |  | MG205510 | MG548193 |
|  | HBG2912 |  | MG205374 | MG548151 |  | MG205508 | MG548194 |
|  | HBG2913 |  | MG205331 | MG548139 |  | MG205467 | MG548183 |
|  | HBG2914 |  | MG205375 | MG548152 |  | MG205468 | MG548195 |
|  | HBG2915 |  | N/A | MG548153 |  | MG205509 | MG548196 |
|  | HBG2916 |  | MG205376 | MG548154 |  | MG205469 | MG548197 |
|  | HBG2917 |  | MG205377 | MG548148 |  | MG205506 | MG548185 |
|  | HBG2918 |  | N/A | MG548155 |  | MG205507 | MG548184 |
| Molnár János | HBG2939 |  | MG205332 | MG548125 |  | MG205482 | MG548199 |
|  | HBG2940 |  | MG205333 | MG548121 |  | N/A | MG548200 |
|  | HBG2941 |  | MG205334 | MG548156 |  | MG205470 | MG548201 |
|  | HBG2942 |  | MG205329 | MG548124 |  | MG205483 | MG548202 |
|  | HBG2944 |  | MG205335 | MG548140 |  | MG205473 | MG548203 |
|  | HBG2945 |  | MG205336 | MG548160 |  | MG205484 | MG548204 |
|  | HBG2946 |  | MG205363 | MG548157 |  | MG205475 | MG548205 |
|  | HBG2949 |  | MG205337 | MG548158 |  | MG205485 | MG548207 |
|  | HBG2950 |  | MG205338 | MG548162 |  | MG205476 | MG548208 |
|  | HBG2951 |  | MG205339 | MG548133 |  | MG205495 | MG548209 |
|  | HBG2952 |  | MG205340 | N/A |  | MG205486 | MG548210 |
|  | HBG2953 |  | MG205341 | N/A |  | MG205490 | MG548211 |
|  | HBG2955 |  | MG205343 | N/A |  | MG205487 | MG548212 |
|  | HBG2956 |  | MG205344 | MG548134 |  | MG205491 | MG548213 |
|  | HBG2957 |  | MG205345 | MG548141 |  | MG205489 | MG548216 |
|  | HBG2958 |  | MG205346 | N/A |  | MG205477 | MG548215 |
| Malom Lake | HBG2979 |  | N/A | MG548135 |  | MG205478 | MG548217 |
|  | HBG2980 |  | MG205360 | MG548130 |  | MG205516 | MG548218 |
|  | HBG2981 |  | MG205361 | MG548161 |  | MG205479 | MG548219 |
|  | HBG2982 |  | MG205347 | MG548128 |  | MG205496 | MG548220 |
|  | HBG2983 |  | MG205348 | MG548129 |  | MG205513 | MG548221 |
|  | HBG2984 |  | MG205349 | N/A |  | MG205514 | MG548222 |
|  | HBG2985 |  | MG205365 | N/A |  | MG205511 | MG548223 |
|  | HBG2986 |  | MG205350 | MG548138 |  | MG205515 | MG548224 |
|  | HBG2987 |  | MG205351 | MG548122 |  | MG205498 | MG548225 |
|  | HBG2988 |  | MG205352 | MG548142 |  | MG205480 | MG548226 |
|  | HBG2989 |  | MG205353 | N/A |  | MG205474 | MG548227 |
|  | HBG2990 |  | MG205354 | N/A |  | MG205481 | MG548228 |
|  | HBG2991 |  | MG205355 | MG548145 |  | MG205488 | MG548229 |
|  | HBG2992 |  | MG205356 | MG548136 |  | MG205492 | MG548230 |
|  | HBG2993 |  | MG205357 | MG548137 |  | MG205500 | MG548231 |
|  | HBG2994 |  | MG205364 | MG548126 |  | MG205497 | MG548232 |
|  | HBG2996 |  | MG205358 | MG548143 |  | MG205494 | MG548233 |
|  | HBG2997 |  | MG205359 | MG548144 |  | MG205472 | MG548234 |
|  | HBG2998 |  | MG205362 | MG548163 |  | MG205493 | MG548235 |
| Dunaalmás, Hungary | 4NH2 |  | N/A | KU948611 |  | KU948711 | N/A |
| Lugovo, Serbia | 2RS5H |  | N/A | KU948616 |  | KU948713 | N/A |
| Šabac, Serbia | 1RS9H |  | N/A | KU948617 |  | KU948714 | N/A |
|  | 2RS9H |  | N/A | KU948618 |  | KU948715 | N/A |
| Freudenau, Austria | 2AU1H |  | N/A | KU948619 |  | N/A | N/A |
| Movila Banului, Romania | 1CR19H |  | N/A | KU948613 |  | KU948712 | N/A |
|  |  |  |  |  |  |  |  |
| Outgroups |  |  |  |  |  |  |  |
| *Niphargus* sp. nov. | HBG2443 |  | MG205517 | MG548164 |  | MG205521 | N/A |
|  | HBG2447 |  | MG205518 | MG548173 |  | MG205522 | N/A |
| *Niphargus forroi* | HBG3990 |  | N/A | N/A |  | N/A | MG548176 |
| *Caecidotea* sp. | Genbank | AF259529.2 | AF259534.1 | AF260834.1 | N/A |  |  |

Table S4. Genealogical Sorting Index estimates for each population of *Asellus aquaticus*. P-values assess significance that exclusive ancestry for each population’s observed is greater than that which would be observed at random.

|  | **Bayesian Phylogeny** | | **Maximum Likelihood Phylogeny** | |
| --- | --- | --- | --- | --- |
| **Population** | ***gsi*** | **p-value** | **gsi** | **p-value** |
| Soroksár (Danube River) | 0.66 | < 0.001 | 0.58 | < 0.001 |
| Balatonfenyves | 0.27 | 0.013 | 0.20 | 0.007 |
| Lipot | 0.22 | 0.016 | 0.18 | 0.009 |
| Cserdi | 0.33 | < 0.001 | 0.24 | 0.003 |
| Malom Lake | 0.93 | < 0.001 | 0.77 | < 0.001 |
| Molnar Janos Cave | 0.39 | < 0.001 | 0.33 | < 0.001 |
| Polgar | 1.0 | < 0.001 | 1.00 | < 0.001 |

Table S5. Genealogical Sorting Index estimates for the three main target populations of *Niphargus hrabei* (outgroup populations removed due to insufficient sample sizes). P-values assess significance that exclusive ancestry for each population’s observed is greater than that which would be observed at random.

|  | **Bayesian Phylogeny** | | **Maximum Likelihood Phylogeny** | |
| --- | --- | --- | --- | --- |
| **Population** | ***gsi*** | **p-value** | **gsi** | **p-value** |
| Soroksár (Danube River) | 0.35 | 0.002 | 0.02 | 0.694 |
| Malom Lake | 0.31 | 0.006 | 0.06 | 0.123 |
| Molnar Janos Cave | 0.23 | 0.088 | 0.18 | <0.001 |
